# Comparative Evaluation of Candida albicans at Two Oral Sites in Healthy Individuals and Individuals With or Without Diabetes With Chronic Periodontitis: A Cross-Sectional Study

**DOI:** 10.7759/cureus.113859

**Published:** 2026-08-03

**Authors:** Muskan Aggarwal, Sheela Gujjari, Sujatha S R

**Affiliations:** 1 Department of Periodontology, JSS Dental College and Hospital, Mysuru, IND; 2 Department of Microbiology, JSS Medical College and Hospital, Mysuru, IND

**Keywords:** candida albicans, chromagar, chronic periodontitis, glycated hemoglobin (hba1c), type 2 diabetes mellitus (dm)

## Abstract

Introduction: There is a synergistic and dysbiotic dental plaque microbial community with a keystone pathogen, *Porphyromonas gingivalis*, initiating the disruption of tissue homeostasis. The pathogenic organisms of periodontitis have been found to interact with other commensal species, such as fungi, of which *Candida albicans* has been found in the subgingival area, adding a new dimension to periodontal pathogenesis. Periodontitis and *Candida* infections are common complications seen in immunocompromised patients such as diabetics.

Aim: This study aimed to detect the presence and distribution of *Candida *from the dorsum of the tongue and subgingival area among healthy controls without periodontitis, patients with diabetes and chronic periodontitis with good or poor glycemic control, and patients without diabetes with chronic periodontitis, and to compare the presence of *Candida *in both sites. This study also aimed to establish an association between *Candida albicans*, probing depth, and glycemic control.

Materials and methods: This was a cross-sectional observational study. It was conducted on 167 patients, and they were divided into four groups, which were as follows: Group A, healthy controls; Group B, individuals without diabetes and with chronic periodontitis; Group C, individuals with diabetes and chronic periodontitis with good glycemic control; and Group D, individuals with diabetes and chronic periodontitis with poor glycemic control. The sample was collected from the dorsum of the tongue with a sterile cotton swab, and subgingival samples were obtained using a Gracey curette in sterile capped containers with phosphate-buffered saline (PBS) and transported to the microbiology department for yeast identification using Sabouraud dextrose agar (SDA) and CHROMagar.

Results: The prevalence of *Candida albicans* on the dorsum of the tongue was 93.1%, and it was statistically significant. (p=0.001), whereas in subgingival plaque it was found to be 62.1%, which was statistically non-significant (p=0.06). The most commonly found *Candida *was *Candida albicans* (53.2%). The other non-albicans species found were *Candida glabrata* (22.9%) and *Candida tropicalis* (23.9%). The highest percentage on the dorsum of the tongue and subgingival plaque was found in diabetic patients with chronic periodontitis with poor glycoregulation (88.1% and 76.2%, respectively). There was a statistically significant association between species abundance and probing depth (p=0.001). A greater number of medium and dense species were found in sites with probing depth > 6 mm. The mean HbA1c with respect to dense species was 9.6±0.7. There was a statistically significant correlation (p=0.002) found between high HbA1c levels and dense candidal species.

Conclusion: Out of the two sampling sites, *Candida albicans* was found more on the dorsum of the tongue. As this was a cross-sectional study, only an association could be established between *Candida*, probing depth, and glycemic control. More longitudinal studies are required to determine *Candida *as the causal organism for the progression of periodontitis and thereby provide antifungal-targeted approaches as a protocol in the future.

## Introduction

In periodontitis, there is a synergistic and dysbiotic dental plaque microbial community with the keystone pathogen *Porphyromonas gingivalis* initiating the disruption of tissue homeostasis [1]. Many studies have reported the presence of subgingival colonization by yeasts, particularly *Candida albicans* [2]. Fungi are found in oral microbiota as both opportunistic pathogens and members of healthy microbiota. *Candida albicans* is the most common among *Candida* species found in the oral cavity, with the ability to change from a commensal to an opportunistic pathogen. The transformation of this commensal microorganism to the pathogenic entity may be linked with factors other than the pathogenic attributes of the organism. This is a unique feature, in contrast to most of the other infections, where the virulence of the organism is considered the principal cause in the pathogenesis. Candidiasis is an opportunistic infection, and an underlying pathology is essential for both the superficial and systemic forms of *Candida *infections. The etiopathogenesis of candidiasis is attributable to three factors: host, fungus, and oral microenvironment-modifying factors. Other non-albican *Candida* species, such as *Candida tropicalis*, *Candida parapsilosis*, *Candida krusei*, *Candida glabrata*, and *Candida dubliniensis*, have also been found in periodontitis. Imbalances in the oral bacterial microbiome due to disturbances or dysfunctions in the host immune system can give rise to oral candidiasis [3]. 

Diabetes has been linked to impaired humoral immunity, neutrophil activity, and T cell responsiveness. Opportunistic infections, including oral candidiasis, are a marker of immunosuppression and an oral symptom of diabetes. Diabetics are more susceptible to candidiasis due to poor glycemic control, xerostomia, smoking, and dentures [4]. The oral cavity is home to the commensal pathogenic yeast *Candida albicans*. The dorsum of the tongue has been identified as the primary oral habitat of Candida albicans, with other sites including mucosa, gingival tissue, dentin, endodontic canals, and periodontal pockets. The incidence of *Candida *in healthy persons has been shown to be 30%-45% [5].

Various studies have contradictory results regarding the mucosal colonization of *Candida albicans* and its presence in periodontal pockets. The presence of the *Candida albicans* species in subgingival areas indicates that subgingival biofilm could be a potential reservoir for these microorganisms. The distribution of *Candida *and non-*Candida *species may vary in the different sites. Depending on ideal factors, the fungal species may thrive more in the subgingival area compared to oral mucosa, which could lead to the progression of periodontitis. Diabetics are more prone to candidal colonization and infection and could contribute to the pathogenesis of chronic periodontitis. Diabetes promotes oral *Candida *colonization by increasing glucose levels and impairing host immunity. Candida albicans adheres to periodontal tissues, forms biofilms with periodontal pathogens, and releases enzymes that damage tissues. It also stimulates pro-inflammatory cytokines, increasing periodontal inflammation, connective tissue destruction, and alveolar bone loss. Thus, *Candida *contributes to the progression and severity of periodontitis in diabetic patients. So, in order to have a global picture of yeast microbiota in the oral cavity, it is necessary to sample oral mucosa and subgingival pockets of patients with and without diabetes.

In the Indian population, there is sparse literature regarding oral candidal carriage in individuals with and without diabetes and the best sampling technique to study the prevalence and distribution of *Candida *at various sites in the oral cavity.

Accordingly, this study aimed to detect the presence and distribution of *Candida *on the dorsum of the tongue and in the subgingival area among healthy controls without periodontitis, individuals with diabetes and chronic periodontitis with good or poor glycemic control, and individuals without diabetes and with chronic periodontitis. The study also compared the presence of *Candida *at both sites and evaluated the association between *Candida albicans*, probing depth, and glycemic control.

The primary outcome was to detect *Candida albicans* from the dorsum of the tongue using cotton swabs and from the subgingival area using a curette in healthy controls, individuals with diabetes and chronic periodontitis, and individuals without diabetes and with chronic periodontitis. The study also aimed to compare the presence of *Candida albicans* between the subgingival area and dorsum of the tongue across all groups.

The secondary outcome was to assess their association with HbA1c levels and periodontal pocket depth.

## Materials and methods

This was a cross-sectional observational study. All participants were recruited from the outpatient department, Department of Periodontology, JSS Dental College and Hospital, Mysuru, India, from March 2024 to March 2025. The study protocol followed for the present study was approved by the Institutional Review Board ethical committee governing the use of human subjects in clinical experimentation at JSS Dental College and Hospital, an affiliated institution of JSS University, Mysuru (IEC no. 37/2024). The clinical trial registry was done for the study with the registry number. (CTRI/2025/12/098546). Written informed consent was obtained from all participants prior to the commencement of the study.

Sample size calculation

The sample size was calculated using the online sample size calculator in JMP version 17 (SAS Institute Inc., Cary, NC, USA). The formula used for analysis was $N = \frac{\left(z_{\alpha} + z_{\beta}\right)^{2} s^{2}}{d^{2}}$

where N = required sample size; zα = z-score for the chosen significance level (α, Type I error/false positive rate); zβ = z-score for the chosen power (β, Type II error/false negative rate); s = standard deviation of the outcome in the population; and d = effect size/minimum detectable difference.

zα = 1.96 (for a 95% confidence level); zβ = 0.84 (for 80% study power); s = 5.5% (standard deviation); d = 2.5 (effect size).

Step 1: $N = \frac{\left(1.96 + 0.84\right)^{2} \times 30.25}{\left(2.5\right)^{2}}$

Step 2: $N = \frac{7.84 \times 30.25}{6.25} \approx 38$ (per group)

Step 3: Total sample size across groups *= 152*

A total of 167 participants participated in the study and were randomly allocated into four groups (Groups A-D). Group A (n=39) consisted of healthy volunteers without clinical signs of chronic periodontitis. Group B (n=39) consisted of healthy subjects diagnosed with chronic periodontitis. Group C (n=47) included patients with type 2 diabetes, patients with good glycoregulation, and diagnosed chronic periodontitis, and Group D (n=42) consisted of patients with type 2 diabetes, patients with poor metabolic control, and diagnosed chronic periodontitis.

Study design and eligibility criteria

Inclusion Criteria

Healthy patients within the age group of 25-35 years, patients with periodontitis within the age group of 35-60 years, and patients with diabetes within the age group of 35-60 years, with a minimum diabetes duration of six months and HbA1c levels ≥6.5%, were included. Individuals with moderate (clinical attachment loss (CAL) of 3-4 mm) to severe generalized chronic periodontitis (i.e., CAL ≥ 5 mm, probing depth ≥ 4 mm in at least 30% of sites, and presence of bleeding on probing) according to the American Academy of Periodontology (AAP, 1999) criteria were selected [6]. Participants with a minimum of 20 teeth were included in the study.

The AAP 1999 Classification [6] was used as it is more familiar to clinicians and practitioners who are not periodontists.

Prior to data collection, the examiner underwent calibration on subjects not included in the study. Intra-examiner agreement was assessed using Cohen's kappa statistic, and a kappa value of 0.87 was obtained, indicating excellent agreement.

Exclusion Criteria

Smokers and pregnant women were excluded from the study. Patients with pre-existing oral candidiasis, denture wearers, those receiving antifungal, antiseptic, antibiotic, or immunosuppressive therapy, and those who had undergone periodontal treatment within the previous 1.5 years were excluded. Patients with diabetes and associated complications such as cardiovascular disease, those receiving anticoagulant therapy, and those with type 1 diabetes were also excluded from the study.

Study protocol

The routine blood investigations and intra-oral X-rays were taken at the first visit. The clinical parameters were recorded. Full mouth clinical examinations were done using a University of North Carolina-15 (UNC-15) probe to measure the pocket depths and CAL.

Group allocation

The patients were grouped into control and study groups, where the control group was sub-grouped into Group A and Group B based on the presence and absence of periodontitis (Table 1).

**Table 1:** Control group allocation

Group	Parameters
Group A	Patients without diabetes and without clinical signs of periodontitis: probing pocket depth (PPD) ≤3 mm, clinical attachment loss (CAL) = 0 mm, and bleeding on probing (BOP) ≤25%.
Group B	Patients without diabetes with moderate (CAL 3–4 mm, PPD ≥4 mm) to severe generalized chronic periodontitis (CAL ≥5 mm, PPD ≥4 mm) affecting at least 30% of sites, with BOP present.

The study group consisted of patients with diabetes and chronic periodontitis. The patients were diagnosed with diabetes by a physician. They were included in the study if they had been diagnosed with diabetes for a minimum duration of six months. Only patients on oral hypoglycemic agents were included, and the latest HbA1c report was considered. Allocation of the study group is presented in Table 2.

**Table 2:** Study group allocation

Group	Parameters
Group C	HbA1c= 6.5-<7.5%
Group D	HbA1c≥7.5%

Collection of samples for detection of *Candida*

Sample Collection

Oral samples were collected from two sites per participant: (1) Dorsum of the tongue: A sterile cotton swab was rubbed across the dorsum of the tongue and placed in a swab tube containing phosphate-buffered saline (PBS). (2) Subgingival sites: Following supragingival plaque removal and site isolation, subgingival samples were collected using Gracey curettes from three sites: site A (<3 mm), site B (4-6 mm), and site C (>6 mm). Each sample was transferred into a vial containing 1 mL of PBS.

Samples were transported to the lab within two hours. The swab cultures were immediately inoculated on Sabouraud dextrose agar (SDA; HiMedia Laboratories Pvt. Ltd., Mumbai, India) and then incubated aerobically at 37°C for 24-48 hours. Yeast identification was performed after 48 hours, and the *Candida *colonies were counted.

*Candida *subspeciation was determined using CHROMagar (HiMedia Laboratories Pvt. Ltd., Mumbai, India). The cultures were inoculated and incubated at 37°C for 24 hours.

Initial Culture on SDA (Quantification)

A 100 µL aliquot from the 1:20, 1:200, and 1:2000 dilutions was spread onto duplicate SDA plates. The plates were incubated at 35-37°C for 24-48 hours.

Colony Enumeration and Preliminary Confirmation

**Figure 1:**
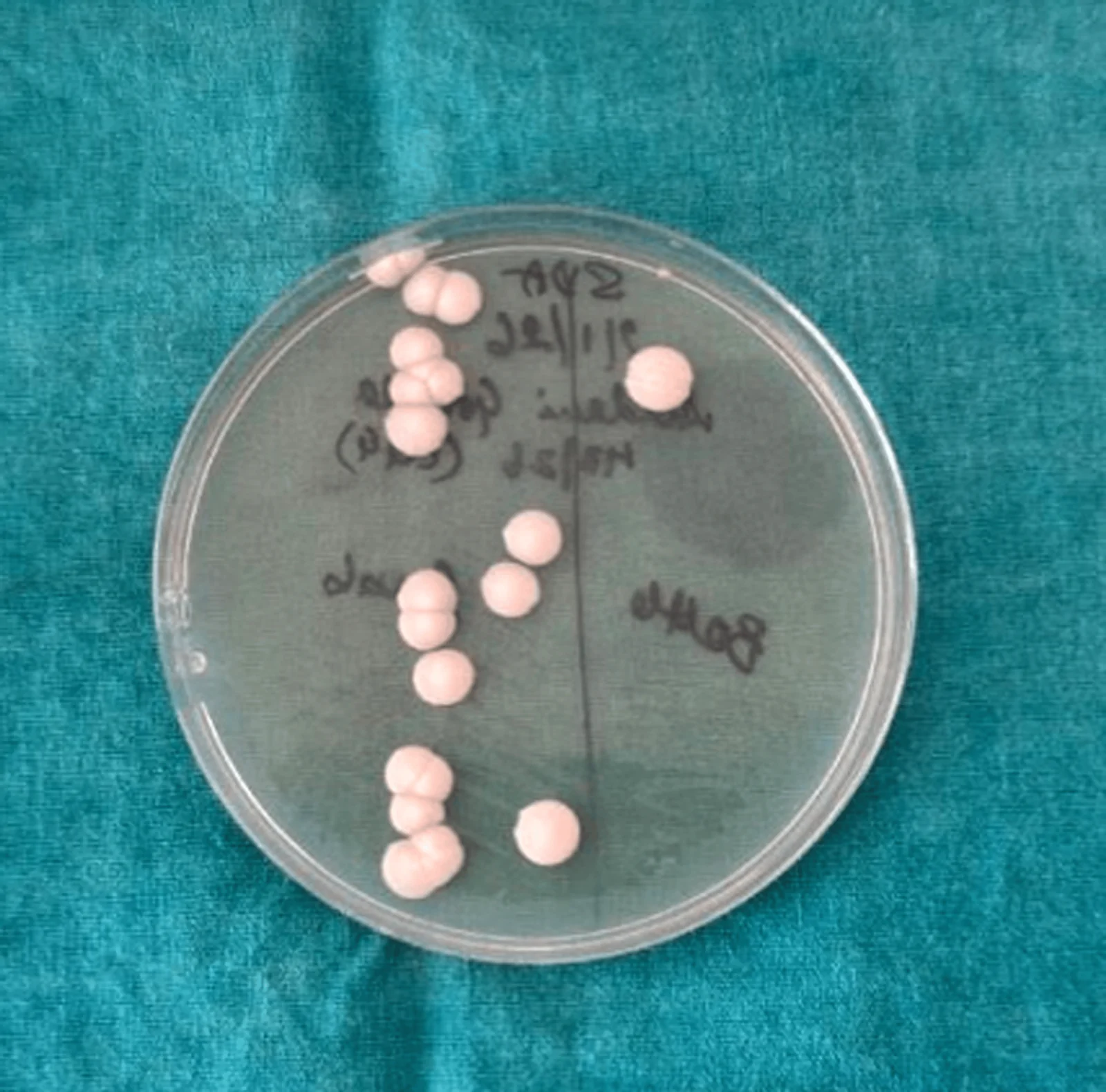
Creamy white colonies of Candida on Sabouraud dextrose agar

Post incubation, creamy white/pasty *Candida*-like colonies (Figure 1) were counted on SDA plates with 30-300 colonies. CFU/ml of original specimen: CFU/ml=Colony count×Dilution factor×10CFU/ml=Colony count×Dilution factor×10 (for example, 30 colonies on 1:200 plate = 30×200×10=60,00030×200×10=60,000 CFU/ml.) 

Tongue dorsum growth was classified as follows: Rare: ≤5 CFU/sample, Medium: 6-50 CFU/sample, Dense: >50 CFU/sample. Subgingival CFU/ml was reported quantitatively (total *Candida *spp.).

Species Identification on CHROMagar

**Figure 2:**
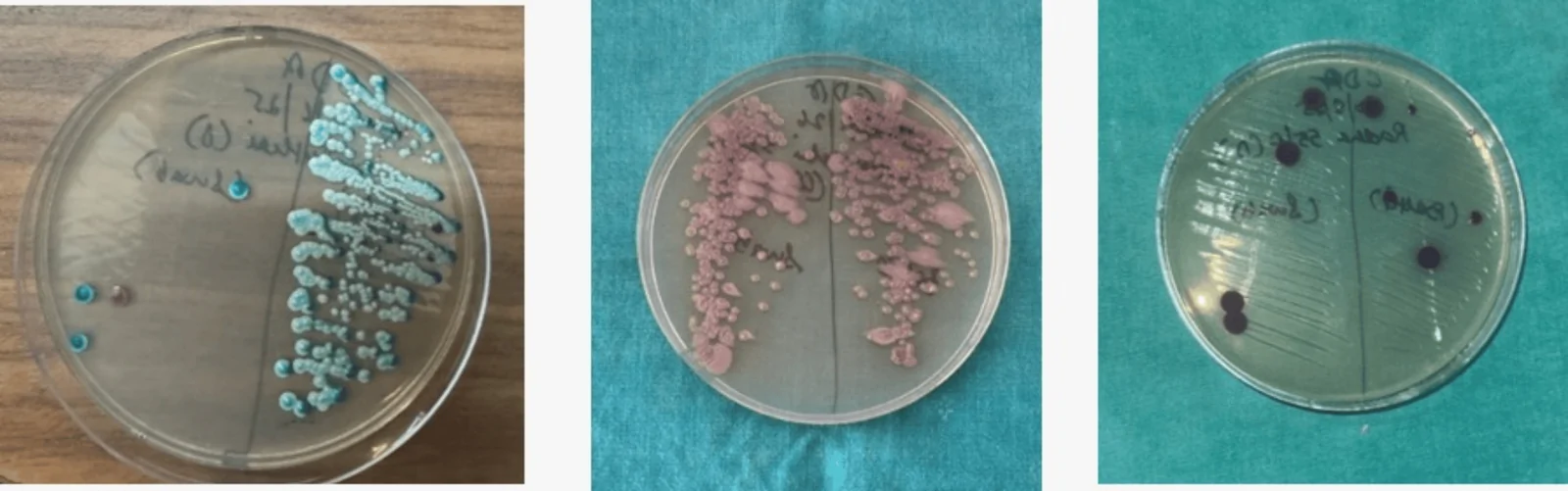
Candida differentiation on CHROMagar (a) *Candida albicans*; (b) *Candida glabrata*; (c) *Candida tropicalis*

Colonies confirmed by smear were subcultured onto CHROMagar Candida plates (e.g., green: *Candida albicans*; blue: *Candida tropicalis*; dark blue-violet: *Candida guilliermondii*). Plates were incubated at 35-37°C for 24-48 hours (Figure 2).

Smear Microscopy 

**Figure 3:**
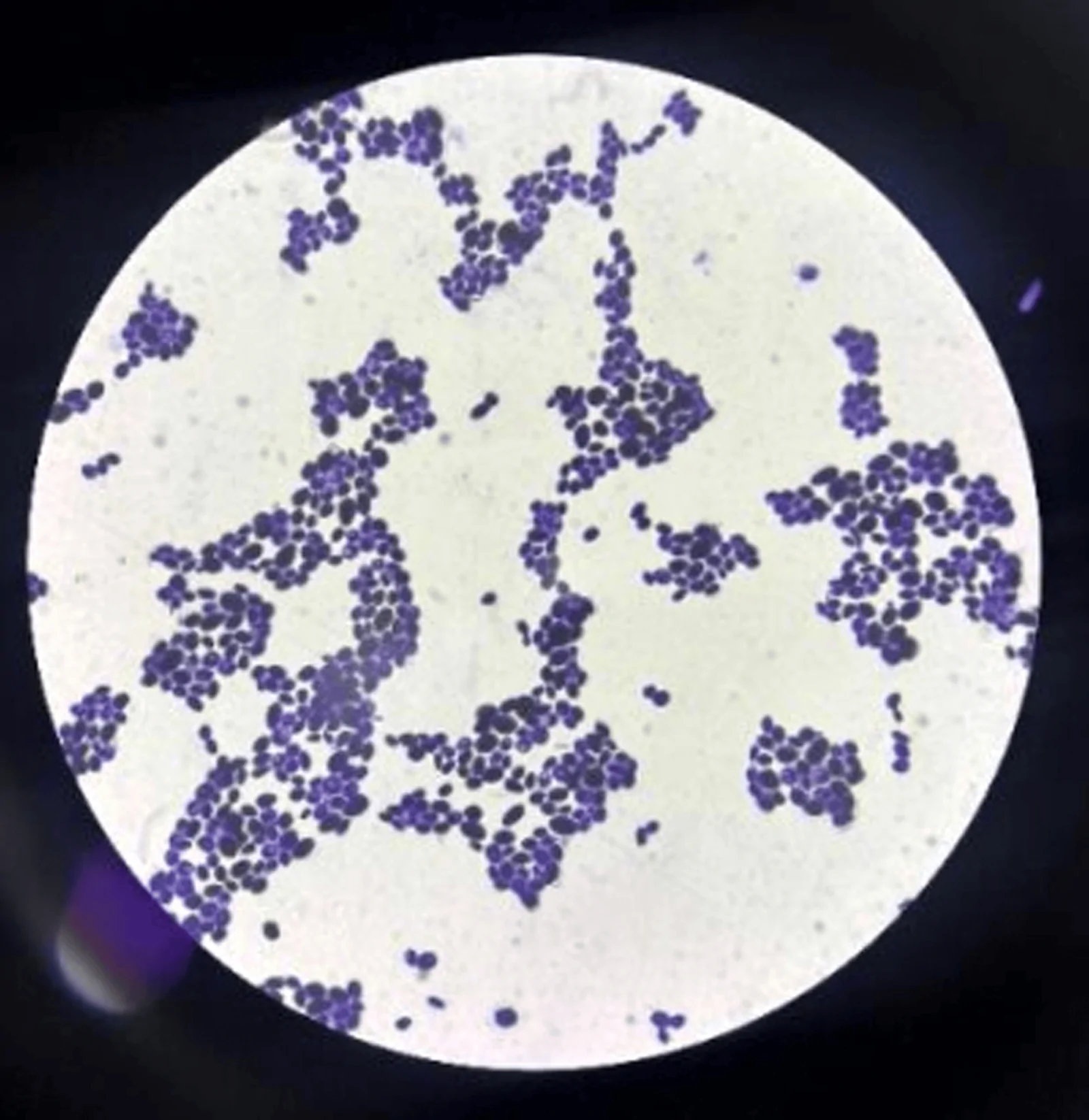
Gram staining was done to confirm the candidal colonies. Bud-like colonies of yeast are seen.

Gram-stained smears from suspicious colonies showed Gram-positive budding yeast cells (oval/round, 2-6 µm), confirming *Candida *morphology (Figure 3).

Blinding

This was a single-blinded study where the microbiologist was blinded.

Statistical analysis

Data were entered into a Microsoft Excel spreadsheet (Microsoft Corp., Redmond, WA, USA) and were analysed using SPSS for Windows version 17.0 (SPSS Inc., Chicago, IL, USA).

Descriptive statistics were used to summarize the study data. Pearson's chi-square test was used to compare the prevalence of *Candida albicans* on the dorsum of the tongue and in the subgingival plaque, to assess the association between *Candida *colonization at the two sampling sites, and to compare *Candida *positivity and the distribution of *Candida *species among the study groups. Spearman's rank correlation analysis was performed to evaluate the relationship between the duration of diabetes and HbA1c levels. The Kruskal-Wallis test was used to compare HbA1c among the three *Candida *growth density categories (rare, medium, and dense). Following a statistically significant Kruskal-Wallis test, post hoc pairwise comparisons were performed using the Mann-Whitney U test. Statistical significance was set at p < 0.05.

## Results

A total of 167 participants who met the inclusion criteria were included in the study, and they were divided into four groups, i.e., Group A (healthy group), Group B (chronic periodontitis group), Group C (patients with diabetes with chronic periodontitis with good glycoregulation), and Group D (patients with diabetes with chronic periodontitis with poor glycoregulation). The data obtained from the measurement of clinical parameters, blood examination, and microbiological assessment were subjected to statistical analysis to obtain the following results.

Among the *Candida*-positive participants (n = 58), *Candida albicans* was isolated from the dorsum of the tongue in 54 (93.1%) participants, while only four (6.9%) participants showed no isolation. The prevalence of *Candida albicans* on the dorsum of the tongue was statistically significant (χ² = 43.103, p < 0.001) (Table 3). In contrast, *Candida albicans* was isolated from the subgingival plaque in 36 (62.1%) participants, whereas 22 (37.9%) participants showed no isolation. This difference was not statistically significant (χ² = 3.379, p = 0.06) (Table 4).

**Table 3:** Frequency of Candida albicans on the dorsum of the tongue Data are represented as N and %. Statistical analysis was performed using the chi-square test (χ² = 43.103). Significance is considered at p < 0.05.

			Candida albicans	p-value	Chi‑square value (χ²)
Dorsum of the tongue	Present	N (%)	54 (93.1%)	<0.001*	43.103
	Absent	N (%)	4 (6.9%)		
Total		N (%)	58 (100.0%)		

**Table 4:** Frequency of Candida albicans in the subgingival plaque Data are represented as N and %. Statistical analysis was performed using the chi-square test (χ² = 3.379). P <0.05 is considered statistically significant

			Candida albicans	p-value	Chi‑square value (χ²)
Subgingival plaque	Present	N (%)	36 (62.1%)	0.06	3.379
	Absent	N (%)	22 (37.9%)		
Total		N (%)	58 (100.0%)		

A significant association was observed between *Candida* colonization of the dorsum of the tongue and subgingival plaque (χ² = 39.568, p < 0.001), with 59 (59.0%) participants showing *Candida *positivity at both sites (Table 5). Comparison of *Candida *positivity among the study groups demonstrated significant differences at both the dorsum of the tongue (χ² = 29.121, p < 0.001) and subgingival plaque (χ² = 40.397, p < 0.001). The highest prevalence of *Candida *positivity at both sampling sites was observed in the poor glycemic control group (88.1% and 76.2%, respectively), while the gingivitis group showed the lowest prevalence (33.3% and 15.4%, respectively). However, site-wise comparisons between the dorsum of the tongue and subgingival plaque within each study group were not statistically significant (Table 6).

**Table 5:** Association of candidal colonization between the subgingival plaque and the dorsum of tongue Data are represented as N and %. Statistical analysis was performed using the chi-square test (χ² =39.568).

			Dorsum of the tongue		Total	p-value	Chi‑square value (χ²)
			Present	Absent			
Subgingival plaque	Present	N (%)	59 (59.0%)	7 (10.4%)	66 (39.5%)	<0.001*	39.568
	Absent	N (%)	41 (41.0%)	60 (89.6%)	101 (60.5%)		

**Table 6:** Comparison of Candida positivity at the dorsum of the tongue and subgingival plaque among the study groups with site-wise comparison Data are presented as n (%). Pearson's chi-square test was used to compare *Candida* positivity among the study groups at each site, while site-wise comparisons between the dorsum of the tongue and subgingival plaque were also performed using Pearson's chi-square test. Statistical significance was set at p < 0.05.

Study group	Dorsum of the tongue n (%)	Subgingival plaque n (%)	Site-wise comparison (p-value)
Gingivitis (n = 39)	13 (33.3)	6 (15.4)	0.923
Chronic periodontitis (n = 39)	27 (69.2)	18 (46.2)	1.000
Good glycemic control (n = 47)	23 (48.9)	10 (21.3)	1.000
Poor glycemic control (n = 42)	37 (88.1)	32 (76.2)	1.000
Chi-square (χ²)	29.121	40.397	NA
p-value	<0.001	<0.001	NA

**Figure 4:**
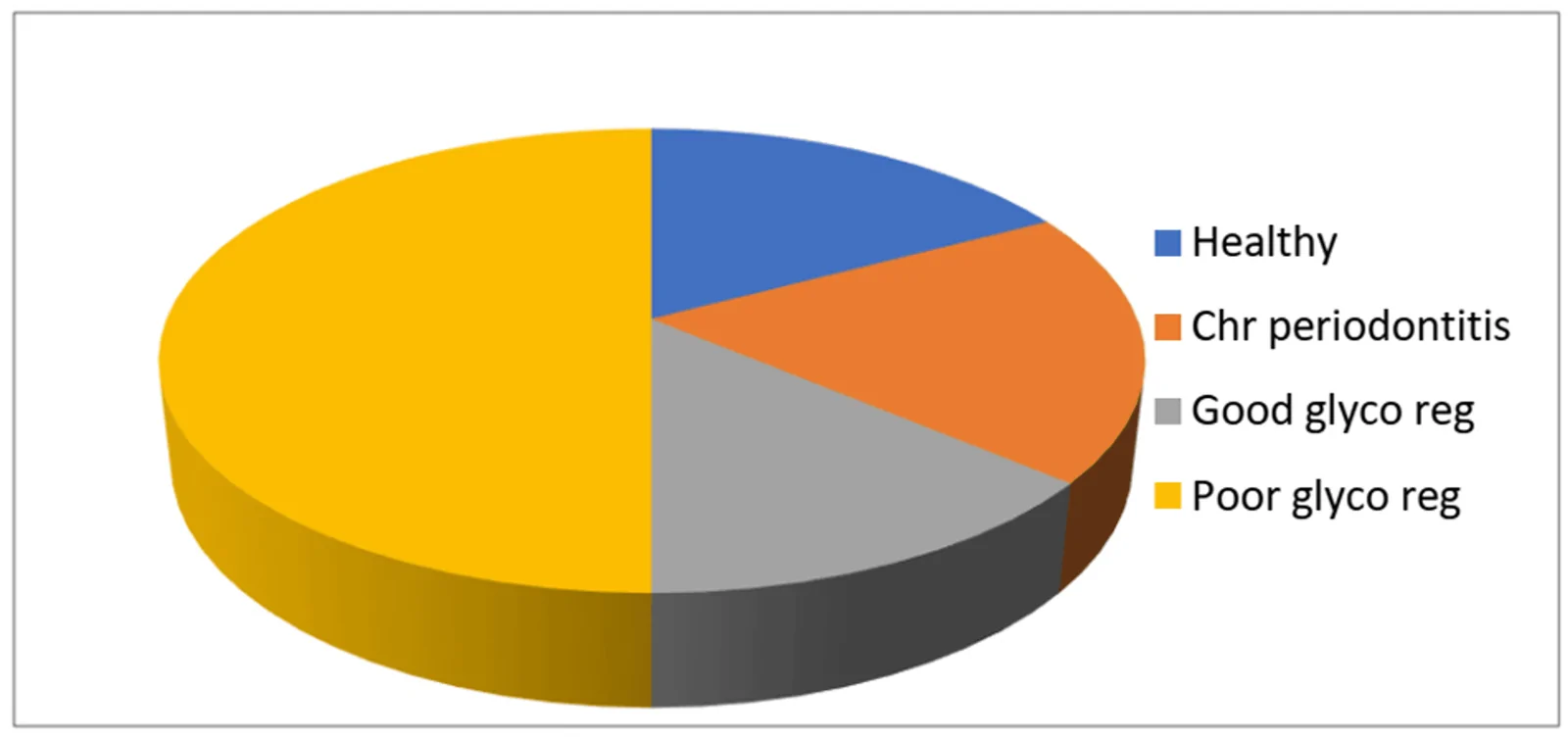
Frequency of Candida albicans among the study groups Chr: chronic; glyco reg: glycoregulation

**Figure 5:**
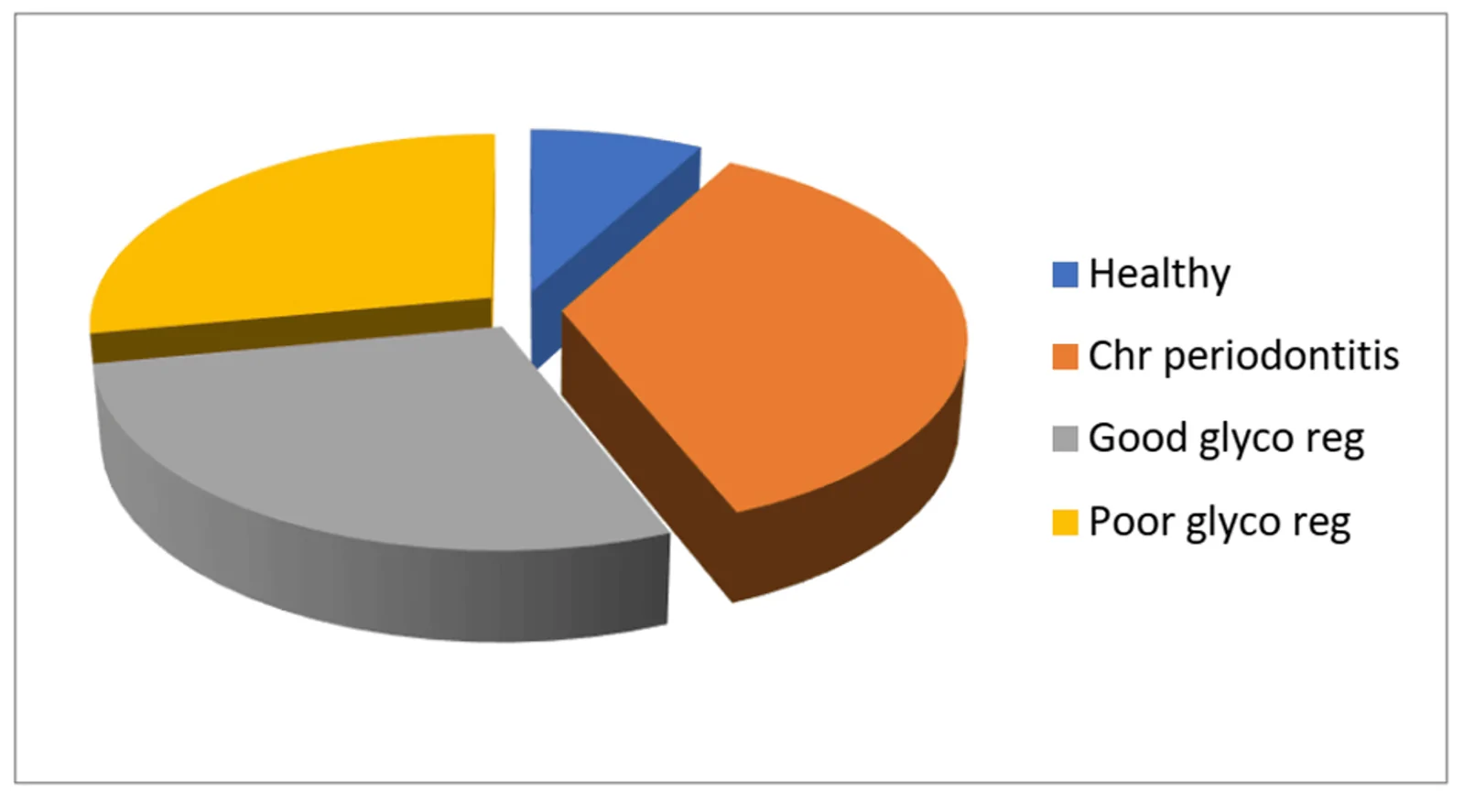
Frequency of Candida glabrata among the study groups Chr: chronic; glyco reg: glycoregulation

**Figure 6:**
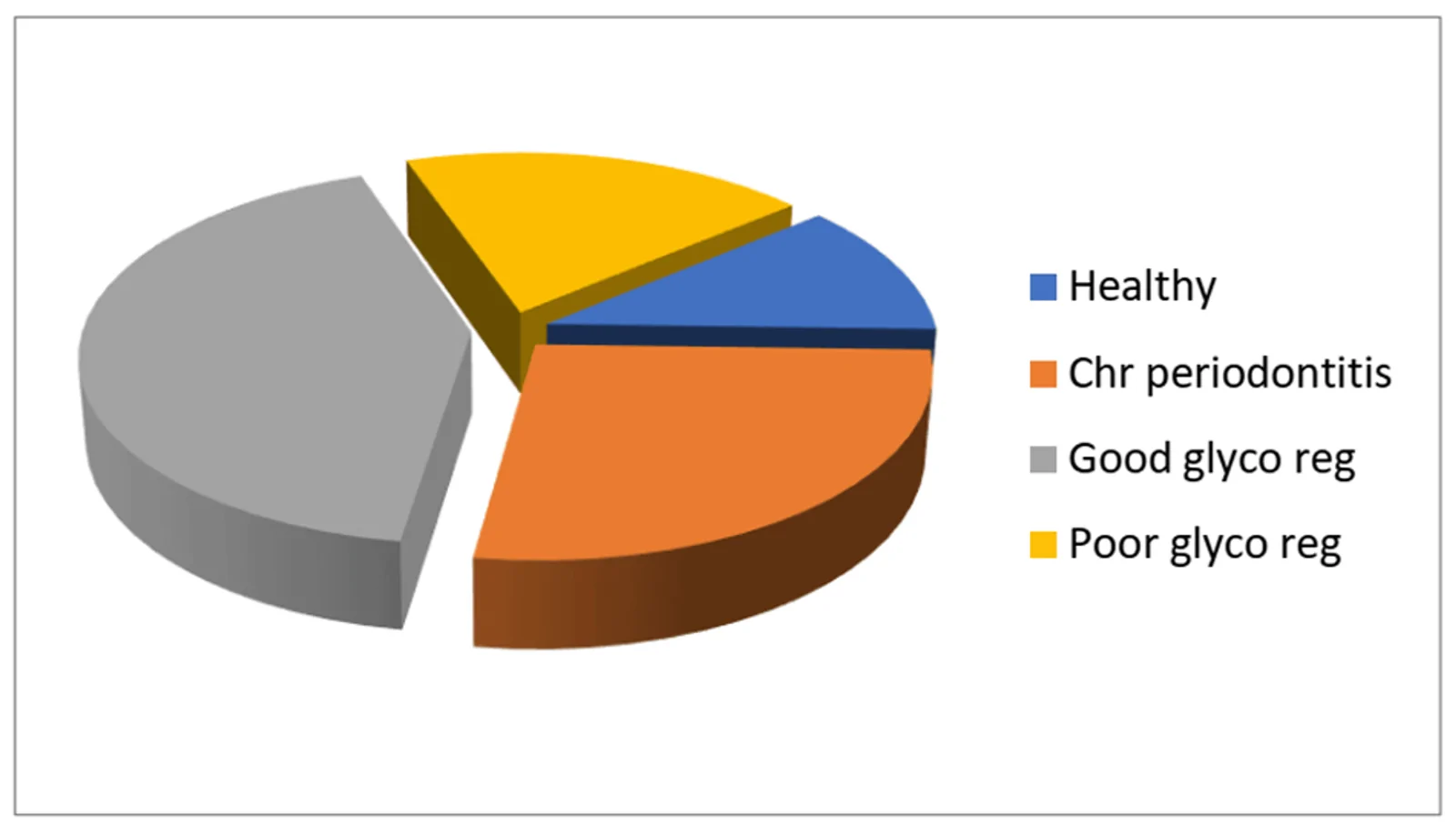
Frequency of Candida tropicalis among the study groups Chr: chronic; glyco reg: glycoregulation

The distribution of *Candida *species differed significantly among the study groups (χ² = 14.986, p = 0.020). Overall, *Candida albicans *was the most frequently isolated species (53.2%), followed by *Candida tropicalis* (23.9%) and *Candida glabrata* (22.9%) (Table 7, Figures 4-6)

**Table 7:** Association between Candida species distribution and the study population Data are represented as N and %. Statistical analysis was performed using the chi-square test (χ² =14.986).

			Healthy	Chronic periodontitis	Good glycoregulation	Poor Glycoregulation	Total *Candida*-positive patients	p-value	Chi‑square value (χ²)
Type of species	Candida albicans	N (%)	10 (66.7%)	11 (40.7%)	8 (30.8%)	29 (70.7%)	58 (53.2%)	0.020*	14.986
	Candida glabrata	N (%)	2 (13.3%)	9 (33.3%)	7 (26.9%)	7 (17.1%)	25 (22.9%)		
	Candida tropicalis	N (%)	3 (20.0%)	7 (25.9%)	11 (42.3%)	5 (12.2%)	26 (23.9%)		

A moderate positive correlation was observed between the duration of diabetes and HbA1c levels (Spearman's ρ = 0.467, p < 0.001), indicating that a longer duration of diabetes was associated with poorer glycemic control (Table 8). 

**Table 8:** Correlation between duration of diabetes and HbA1C Data are presented as Spearman's rank correlation coefficient (ρ). Correlation between duration of diabetes and HbA1c was assessed using Spearman's rank correlation. A p-value < 0.05 was considered statistically significant.

Variable 1	Variable 2	Spearman's rho	p-value
Duration of diabetes(years)	HbA1c (%)	0.467	<0.001

Comparison of HbA1c according to *Candida *growth density revealed a significant difference among the three growth categories (Kruskal-Wallis χ² = 9.593, p = 0.002). The mean HbA1c increased progressively from the rare growth group (7.08 ± 0.62%) to the medium (8.09 ± 1.21%) and dense growth groups (9.68 ± 0.78%). Post hoc pairwise comparisons using the Mann-Whitney U test demonstrated significant differences between rare and medium growth, rare and dense growth, and medium and dense growth groups, indicating that increasing *Candida *growth density was associated with poorer glycemic control (Table 9).

**Table 9:** Correlation between glycemic control (Hba1c) and Candida species density Data are presented as mean ± standard deviation (SD). Overall group comparison was performed using the Kruskal–Wallis test. Post hoc pairwise comparisons were performed using the Mann–Whitney U test. A p-value < 0.05 was considered statistically significant. p < 0.05.

*Candida *growth	n	Mean HbA1c ± SD	p-value	Chi-square value
Rare	19	7.08 ± 0.62	0.002*	9.593
Medium	31	8.09 ± 1.21		
Dense	16	9.68 ± 0.78		

**Figure 7:**
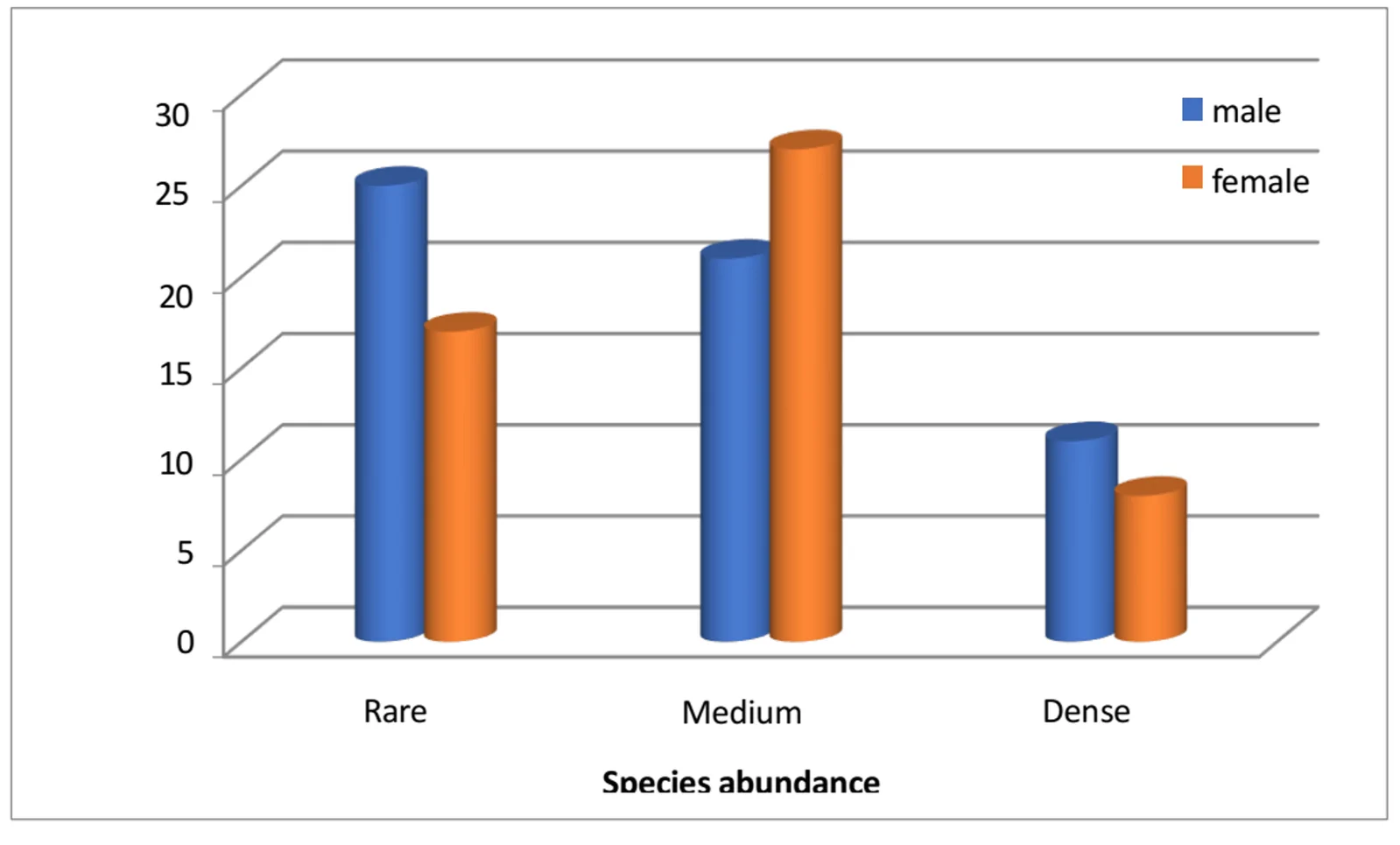
Correlation of number of candidal species with gender

The total number of males and females in the study was 52.7% and 47.3%, respectively. There was no statistical difference established between the groups on the basis of gender. When the number of *Candida* species was correlated with gender, there was no significant difference found between males and females (Figure 7).

**Figure 8:**
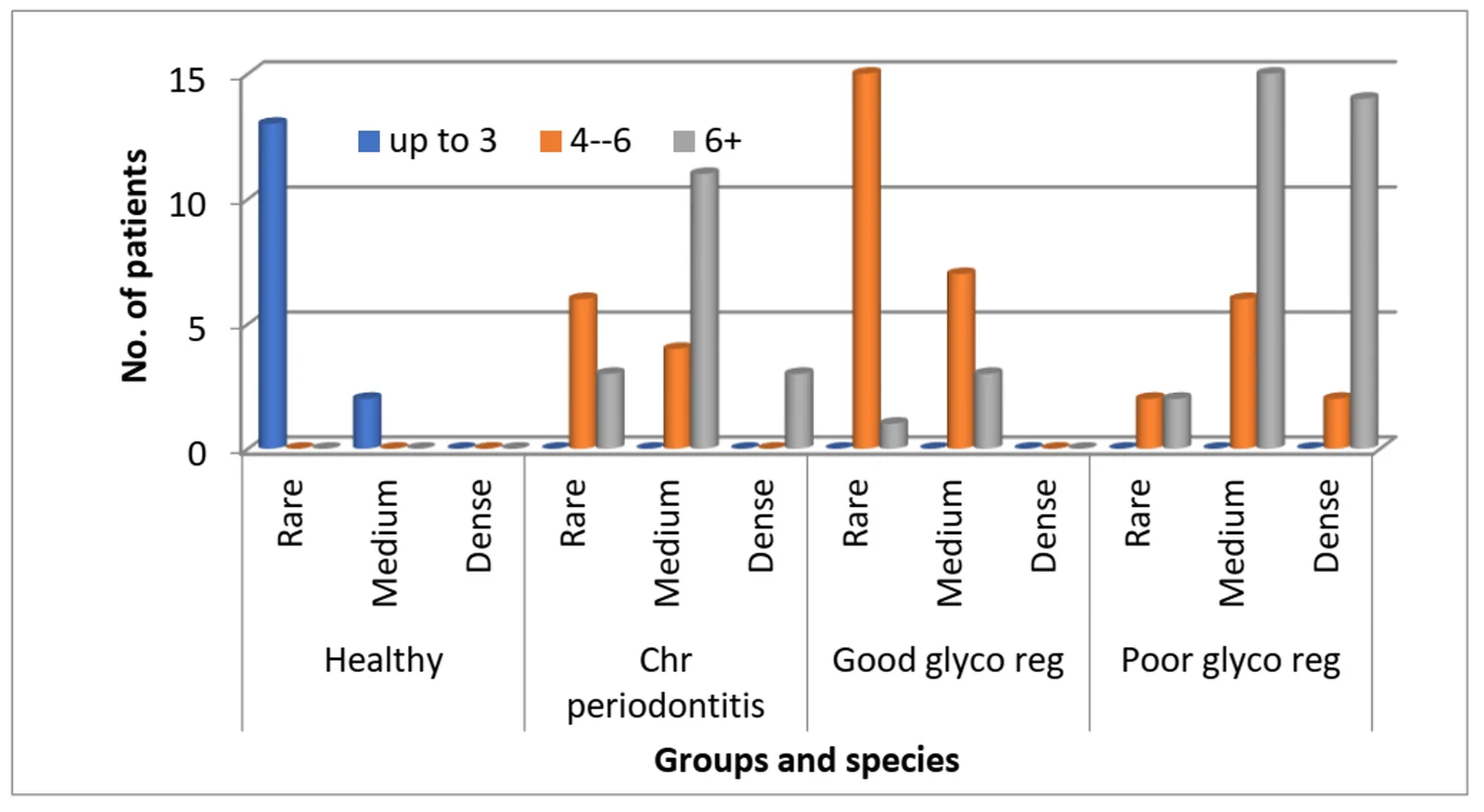
Corelation of number of candidal species with periodontal probing depth among study population

There was a statistically significant association between species abundance and probing depth. More number of medium and dense species were found in sites with probing depth > 6 mm (Figure 8).

## Discussion

More than 700 species of bacteria have been found in the oral cavity. Some of these are beneficial, and some are harmful. So, proinflammatory characteristics and interspecies signaling result in community shifts in microbiota along with host responses, leading to periodontal destruction. The presence of other commensal organisms like fungi in the oral cavity has kindled a new insight into the pathogenesis of periodontitis, of which *Candida albicans *has been found to be the most prevalent.

Diabetes and periodontitis share a bidirectional relationship. The dysregulated immune system plays a main role in the pathogenesis of diabetes and its associated complications. It is characterized by a persistent inflammatory state and an impaired ability to resolve the immune response. Hyperglycemia, xerostomia, alteration in the microflora, and altered collagen metabolism predispose individuals to oral candidiasis [4].

*Candida *species are found to reside on mucosa, the tongue, and saliva and have also been isolated from periodontal pockets [7]. Hence, this was a cross-sectional observational study of one year's duration conducted to analyze oral candidal carriage from the dorsum of the tongue and subgingival area of patients with chronic periodontitis with and without diabetes and to correlate the findings with glycemic levels.

In our study, *Candida *species were found on the dorsum of the tongue in the healthy group, patients with chronic periodontitis without diabetes, patients with diabetes and good glycemic control, and patients with diabetes and poor glycemic control; these percentages were 33.3%, 67.5%, 52%, and 89.5%, respectively. The group-wise comparison of the presence of *Candida *species on the dorsum of the tongue showed statistically significant results (p = 0.001). The highest percentage was observed among patients with diabetes and chronic periodontitis with poor glycemic control (89.5%). This finding was in accordance with other studies conducted by Khosravi et al., Sardi et al., and Belazi et al., who also reported a higher prevalence of *Candida *species on the tongue among patients with diabetes [8-10].

The *Candida *species present in the subgingival plaque in the healthy group, chronic periodontitis group, diabetic patients with good glycoregulation, and poor glycoregulation were 15.4%, 47.5%, 24%, and 76.3%, respectively. When the group-wise comparison was done for the presence of *Candida *species in the subgingival plaque, the results were found to be statistically significant (p=0.001). The highest percentage was found in diabetic patients with chronic periodontitis with poor glycoregulation (76.3%). The results were similar to the study done by Petrovic et al. [11] and Odds et al. [12]. This could be due to the microvascular alterations and elevated glucose concentration in saliva and gingival crevicular fluid, which leads to decreased salivary pH, which increases the growth of acidogenic microorganisms like *Candida albicans* [13]. In all the groups, candidal species were found in the subgingival area with the predominance of *Candida albicans*, which could imply that the subgingival area can be a reservoir and could contribute to the pathogenesis of periodontitis. This could be explained by their ability to adhere to a non-shedding hard surface characterized by distinct primary colonizers and variations in redox potential, pH, electrochemical potential, and nutrient composition, along with differing oxygen availability, which could make it more favorable for colonizing in the subgingival area [11]. Controversial results were reported by Petrović et al., where they found no statistical difference in subgingival yeast presence among diabetics and non-diabetics with chronic periodontitis. This could be because of the use of two sampling methods for subgingival plaque collection [14].

The null hypothesis was that there is no significant difference in candidal carriage on the dorsum of the tongue and subgingival plaque in patients who are diabetics and non diabetics with chronic periodontitis. Also. there is no correlation between glycemic levels and *Candida *species colonization.

In this study, *Candida *species were detected on the dorsum of the tongue (59.9%) and in the subgingival area (39.5%) among healthy individuals, patients with diabetes, and patients with chronic periodontitis without diabetes. The association among them was statistically significant (p=0.001), as a greater number of participants presented *Candida *species on the dorsum of the tongue than in subgingival plaque. This is in accordance with the study done by Radunovic et al. [7]. The ability of *Candida *to adhere to epithelial cells and mucosal cells might be the reason for its prevalence more on the dorsum of the tongue. Another reason could be attributed to relatively lower subgingival plaque collection when compared to the sample obtained from the dorsum of the tongue.

In our study, we found that when *Candida *species were present on the dorsum of the tongue, a greater number of participants had *Candida *species present in the subgingival plaque as well. But when the *Candida *species was absent on the dorsum of the tongue, only a few participants showed candidal growth in the subgingival plaque. In a study done by Gayathri et al., it was shown that specific *Candida *species obtained in saliva samples were not seen in subgingival plaque samples of the same subject [15]. This shows that individual sites are colonized by distinct *Candida *species (site-specific), which are otherwise not seen on oral mucosa or pockets in healthy patients. This stresses the fact that standardization of sampling techniques is based on sites that harbor more *Candida*. Hence, this study was an attempt to check the distribution of *Candida*.

*Candida albicans* is the most commonly found species in the oral cavity, but other non-albicans species such as *Candida tropicalis*, *Candida parapsilosis*, *Candida glabrata*, *Candida auris*, *Candida guilliermondii*, and *Candida krusei* have also been found [16]. *Candida *can colonize various sites in the oral cavity, such as the dorsum of the tongue, buccal mucosa, subgingival area, dentin, and endodontic canals [17]. In our study, the total number of *Candida albicans* found in all groups was 53.2%. The total number of *Candida glabrata *found in all groups was 22.9%. The total number of *Candida tropicalis* found in all groups was 23.9%. Thereby, a statistically significant association was found between *Candida *species and study groups. However, *Candida albicans* was predominantly found (53.2%), followed by *Candida glabrata* and *Candida tropicalis*. This is in accordance with other studies [18, 19]. *Candida albicans* switches between yeast and hyphal forms, which enables its invasion into tissues and the immune system and also biofilm formation. The ability of *Candida albicans* to react to a wide range of environmental imbalances, including variations in nutrition, temperature, and pH, and its complex genetics because of its virulence genes, makes it more pathogenic than other species, and that is why it is found more [13]. There were other studies that found *Candida dubliniensis* as well along with *Candida albicans* [20]. In the study done by Urzua et al., it was seen that *Candida albicans* and *Candida dubliniensis* were capable of colonizing the periodontal pockets in patients with chronic periodontitis, while only *Candida albicans* was isolated from the subgingival area of healthy subjects and patients with aggressive periodontitis [17].

The prevalence of *Candida albicans* on the dorsum of the tongue was 93.1%, and it was statistically significant. (p=0.001), whereas in subgingival plaque, it was found to be 62.1%, which was statistically non-significant (p=0.06). This was in accordance with studies done by Petrovic et al. in 2015 and 2019, where they also found a higher percentage of *Candida albicans* on the dorsum of the tongue [11, 14].

There are various techniques available for the isolation of *Candida *within the oral cavity, such as smear examination, plain swabs, imprint culture, collection of whole saliva, concentrated oral rinse, sterile paper points or curettes for subgingival plaque sampling, and mucosal biopsy [21]. However, there is limited literature regarding the most appropriate method for *Candida *isolation. Therefore, in this study, two techniques were used: cotton swabs were used to collect samples from the dorsum of the tongue, and sterile curettes were used to collect subgingival plaque. Sterile curettes collect a greater total amount of biofilm, including tightly adhered microorganisms, and provide access to deeper periodontal pockets. Paper points are more suitable for the collection of gingival crevicular fluid and may not reach the depth of deep pockets because they become saturated before reaching the base of the pocket [22]. Since *Candida* spp. form colonies on the surface of epithelial cells, it is necessary to gently "scratch" the surface using a curette to improve the accuracy of detection [14]. Additionally, differences in paper point size and insertion duration among studies may influence subgingival plaque sampling outcomes.

Glycemic control in patients with diabetes plays an important role in the progression of periodontal diseases and vice versa. Studies have shown that patients with diabetes who have good glycemic control experience relatively less periodontal destruction than those with poor glycemic control. Additionally, *Candida *species have been reported more frequently in patients with diabetes and poor glycemic control [2]. In our study, the presence of *Candida *species on the dorsum of the tongue was higher among patients with poor glycemic control (88.1%) compared with those with good glycemic control (48.9%). Similarly, the presence of *Candida *species in subgingival plaque was higher among patients with poor glycemic control (76.2%) than among patients with good glycemic control (21.3%). Our results were consistent with the study conducted by Mubarak et al., who concluded that good glycemic control provides better protection against *Candida albicans* colonization [20]. Other studies have also demonstrated that maintaining adequate blood glucose levels reduces the risk of candidal infections [23-25]. However, the findings of this study differed from those of Melton et al., who reported no association between glycemic control and the presence or absence of *Candida *species [26].

In our study, a moderate positive correlation was observed between the duration of diabetes and HbA1c levels (Spearman's ρ = 0.467, p < 0.001), indicating that longer duration of diabetes was associated with poorer glycemic control. This was in accordance with a study done by Verma et al where HbA1c levels showed a significant increase with the duration of diabetes [27]. This research implies that a longer duration of diabetes may affect glycemic regulation, presumably as a result of increased insulin resistance and growing β-cell malfunction [28]. Also long time consumption of sugar, genetics, and lifestyle modification were suggested [29].

There was a statistically significant correlation (p=0.002) found between high HbA1c levels and dense candidal species. The results were similar to the studies done by Lipsa et al where they observed higher CFU/ml in the patients with poorly controlled diabetes [30].

There was a statistically significant association between species abundance and probing depth(p=0.001). A greater number of medium and dense species were found in sites with probing depth > 6 mm. However, for the individual groups, no significant association was seen. This was in accordance with studies done by Torre et al and Canabarro et al [2, 31]. The reason given is that it has high pathogenic potential due to its different properties and virulence factors, which allow it to remain in the periodontal pocket, thereby evading elimination through oral hygiene practices [2]. There are reports of the presence of hyphae on the border of sulcular epithelium in subepithelial connective tissue, which results in additional tissue invasion as well as co-aggregation with microbial species, leading to progression of chronic periodontitis. Also, it may form a barrier that prevents access of anti-microbial molecules and immunoglobulins present in the oral cavity, thereby blocking the therapeutic action of antibiotics [32]. The results were not in accordance with the study done by Urzua et al where they aimed to determine if the depth of the pocket favoured the development of yeast, and they found that the degree of colonization is not related to the depth of the pocket [17].

The study had some limitations. Presence of other red complex bacteria could have been studied for the relationship between *Porphyromonas gingivalis* and *Candida*. Most of the studies have used saliva samples for isolation of *Candida*. This could have been used in comparison with subgingival plaque. Better quantification methods could have been used for assessing *Candida*. The 2017 classification could have been used for classifying the subjects according to periodontal status. Antibiotics could have been incorporated in SDA by suppressing the growth of other bacterial species and promoting the growth of *Candida*. The confounders such as age, oral hygiene, diet, salivary flow, xerostomia, and medications were not taken into account. So there was a lack of multivariable adjustment for confounders. *Candida *confirmation with other methods could have been included. Possible selection bias could have been present, and also there was an unequal age range among healthy and periodontitis groups. It was a single-centre study, which is cross-sectional. A causal relationship between the prevalence of *Candida *and periodontitis, as well as the prevalence of *Candida *and poor glycemic control, could not be established. It could be attributed only to an association.

## Conclusions

*Candida *was isolated more frequently from the dorsum of the tongue than from the subgingival plaque, suggesting that the dorsum of the tongue may serve as a more accessible and potentially reliable sampling site for the detection of *Candida albicans*. However, further longitudinal studies are required to validate its diagnostic utility. In addition to *Candida albicans*, *Candida glabrata *and *Candida tropicalis* were also isolated, indicating the presence of non-albicans Candida species in the oral cavity. Greater *Candida *growth density was observed at sites with probing depths greater than 6 mm, suggesting that deeper periodontal pockets may provide a favorable environment for *Candida *colonization. The highest prevalence of *Candida *colonization was observed in diabetic patients with chronic periodontitis and poor glycemic control, highlighting the possible influence of hyperglycemia on oral *Candida *colonization. As the present study employed a cross-sectional design and did not account for all potential confounding factors, only an association could be established, and a causal relationship could not be inferred. Therefore, future longitudinal studies with larger sample sizes, inclusion of relevant confounding variables, and molecular identification techniques are recommended to further elucidate the role of *Candida *species in periodontal disease and diabetes.
